# Legislation for Midwives

**Published:** 1900-05-19

**Authors:** 


### The Institutional Workshop.

### LEGISLATION FOR MIDWIVES.

By a Correspondent.

(*Continued from page 436, Vol. XXVII.*)

### III.—THE CONSTITUTION OF THE CENTRAL BOARD.

In a previous article it was shown that legislation, however useful from a disciplinary point of view, has not in other countries succeeded in procuring a high standard of midwifery.

It will be well to examine what steps have been taken by the promoters of the Bill now under consideration by Parliament to ensure that the high standard at present existing in this calling in England shall not be suffered to decline.

The main feature of the Midwives Bill is the establishment of the Central Midwives Board. Every point of vital importance is practically left to the discretion of this all-powerful little committee. The Central Midwives Board is to consist of seven persons. Four are to be medical practitioners appointed respectively by the Royal College of Physicians of London, the Royal College of Surgeons of England, the Society of Apothecaries, and the Incorporated Midwives Institute. Two persons (one of whom shall be a woman) are to be appointed by the Lord President of the Council, and one person by the Association of County Councils. This Central Board is to be charged with the forming of rules regulating the issue of certificates and the conditions of admission to the Midwives' Roll; they are to regulate the course of training and the conduct of examinations, and in their hands alone (subject to the approval of the General Medical Council) will rest the establishment of a due standard of education for midwives.

It is a strange omission which excludes from the members of this Board any representation of the great training schools for midwives which, as I have pointed out, have been efficiently grappling with the real problems involved for over a century. If the Central Board is to have power over the issuing of certificates and the appointment of examiners, it is clear that their decisions will affect very materially the course of instruction given at all the training schools. And it will hardly be deemed satisfactory that the curriculum at these centres, where scientific midwifery has seen its rise, and whence it has spread to all parts of the kingdom, should now be placed practically under the complete control of seven persons, no one of whom is required to possess any special knowledge in the examination or training of the class of persons chiefly concerned. Who is to ensure that the bodies charged with the duty of selecting representatives for this Central Board will not be guided more by a sounding name than by the practical qualifications of their delegate? It is quite possible for a medical man to be a first rate accoucheur, with a reputation which immediately commands respect, and yet to have the very haziest ideas as to the amount of knowledge of the craft which can be put into an ignorant woman in a given period. All who have any experience of examinations are well aware of the facility with which a high sounding list of subjects can be strung together, and of the fatal effects of attempting to force upon half-educated candidates a smattering of science. But it takes years of patient wrestling with successive batches of pupil midwives before an examiner or instructor can get a clear grasp of the essential points to enforce, and of what is best omitted altogether. It is of the utmost importance that some of those serving on the Central Midwives' Board should have had this practical experience in a training school for midwives, and I cannot think that this Board will command confidence from experts until the midwifery schools are accorded representation at its councils.

I believe it to be due in part to the entirely practical character of the training of midwives, and the absence of any pretentious code made compulsory upon all candidates for instruction that the results have been so satisfactory in the past. English midwives have won for themselves a high place in comparison with those of other countries, and I cannot but fear lest with the introduction of a centralised system there may insensibly follow a substitution of theory for practice and a straining after effect which shall impair the soundness of the knowledge imparted. The only safeguard would seem to be the presence of experienced practical men skilled in this highly technical branch of teaching, on the Board, and this of necessity and not of choice.

By far the most responsible and wide-reaching of the duties of the Board is this vital matter touching the standard of midwifery to be maintained. The provisions of the Bill relating to the regulation of the midwives' calling are in most respects admirable. There will be a midwives' roll in which for a fee of one guinea midwives who desire to practise and whose qualifications satisfy the Board must have their names inscribed, and each midwife must in addition procure a licence to practice in her county or borough. The licence is to be granted by the local supervising officer, and as the prescribed fee is but one shilling there will practically be no difficulty in a midwife taking cases where she will.

The disciplinary powers of the Board are among the most necessary and wholesome provisions of the Bill, and it is a wise safeguard against local jealousy and animosity that the Central Board is made the final court for charges of malpractice, negligence or misconduct on the part of any midwife whose name it is sought to remove from the roll.

I propose in a further article to consider more particularly the manner in which this Bill is likely to affect midwives and midwifery in general.

